# Highly Efficient and Stable Quantum Dot Light-Emitting Diodes Employing Sputtered SnO_2_ Layer as Electron Transport Layers

**DOI:** 10.3390/nano16010031

**Published:** 2025-12-25

**Authors:** Jaehwi Choi, Jiwan Kim

**Affiliations:** Department of Advanced Materials Engineering, Kyonggi University, Suwon 16227, Republic of Korea

**Keywords:** QLEDs, sputtering, ETL, SnO_2_

## Abstract

We report a novel approach to fabricating high-performance and robust quantum dot light-emitting diodes (QLEDs) utilizing sputtered SnO_2_ thin films as the electron transport layer (ETL). While conventional solution-processed ZnMgO NP ETLs face limitations in mass production, the sputtering process offers advantages for uniform and reproducible thin film deposition. Herein, the structural, optical, and electrical properties of SnO_2_ thin films were optimized by controlling the Ar/O_2_ ratio and substrate heating temperature during sputtering. SnO_2_ thin films with O_2_ gas improve charge balancing in QLEDs by lowering the conduction band minimum. Furthermore, it was observed that oxygen vacancies in SnO_2_ function as exciton quenching sites, which directly impacts the long-term stability of the device. QLEDs fabricated under optimal conditions (Ar/O_2_ = 35:5, 200 °C heating) achieved a peak luminance of 99,212 cd/m^2^ and a current efficiency of 21.17 cd/A with excellent device stability. The findings suggest that sputtered SnO_2_ ETLs are a highly promising technology for the commercial production of QLEDs.

## 1. Introduction

Colloidal quantum dots (QDs) are semiconductor nanocrystals that have emerged as highly promising materials for next-generation display technologies. Owing to the quantum confinement effect, their emission wavelength can be precisely tuned by controlling their size, exhibiting exceptional color purity with a narrow full-width at half-maximum [1,2,3,4]. These unique optoelectronic properties have propelled the development of quantum dot light-emitting diodes (QLEDs), which have demonstrated remarkable performance advancements since their first report in 1994 [5,6,7,8]. Through the optimization of QD core/shell structures and the engineering of charge transport layers, recent advancements in QLED performance, characterized by external quantum efficiencies exceeding 22%, have solidified their status as a benchmark technology for the upcoming era of lighting and displays [9].

The performance of a QLED is critically dependent on the efficient injection and transport of charge carriers into the emitting layer (EML). To this end, inorganic metal oxide nanoparticles (NPs), particularly ZnMgO, have been extensively employed as electron transport layers (ETLs), replacing conventional organic materials [10,11,12]. Solution-processed ZnMgO NP ETLs offer suitable energy level alignment with QDs and facile fabrication, which has led to significant improvements in device luminance and efficiency. However, the solution-based spin-coating method presents inherent challenges for commercial viability. Residual hydroxyl and acetate ligands on the NP surface can act as charge traps, impeding electron transport, while the aggregation of NPs in colloidal solutions often compromises the uniformity and reproducibility of the film, especially over large areas [13,14].

To overcome these limitations, SnO_2_ has been identified as a superior alternative ETL material to ZnMgO. SnO_2_ exhibits a deeper conduction band minimum, which can lower the electron injection barrier from the ETL to the QDs, and possesses comparable or even higher electron mobility, alongside greater chemical stability [15,16]. Therefore, QLEDs utilizing solution-processed SnO_2_ NPs as an ETL have demonstrated enhanced performance, confirming the material’s intrinsic advantages [17,18]. Nevertheless, these devices are still constrained by the same fabrication issues associated with the solution-based deposition of NP films.

An ideal approach to resolve these issues is to replace the solution-based process with a vacuum deposition technique. Sputtering, a well-established physical vapor deposition method, is renowned for its ability to deposit highly uniform, dense, and reproducible thin films over large-area substrates, making it highly compatible with mass production. While there have been reports on sputtered ZnO or ZnMgO ETLs for QLEDs, their device performance has often lagged behind that of their best solution-processed counterparts [19,20]. To date, however, the application of sputtered SnO_2_ as an ETL in QLEDs has not been reported yet.

Herein, we demonstrate high-performance and durable QLEDs by employing a sputtered SnO_2_ thin film as the ETL, thereby synergistically combining the material advantages of SnO_2_ with the process advantages of sputtering. We systematically investigated the structural, optical, and electrical properties of the sputtered SnO_2_ films by varying Ar/O_2_ ratio and substrate heating temperature. QLEDs using SnO_2_ ETL deposited under an optimized Ar/O_2_ ratio of 35:5 reached a maximum luminance of 99,212 cd/m^2^ and current efficiency of 21.17 cd/A after heating at 200 °C, representing one of the highest performances for QLEDs employing SnO_2_ as the ETL. The optimized SnO_2_ ETL enabled efficient electron injection and transport, leading to QLEDs with significantly improved luminance, current efficiency, and operational stability. This work highlights the potential of sputtered SnO_2_ as a robust and effective ETL for the fabrication of commercially viable QLEDs.

## 2. Materials and Methods

### 2.1. Synthesis of Green QDs

To synthesize green CdZnSeS/ZnS QDs, CdO (0.14 mmol) and ZnO (3.41 mmol) were combined with 7 mL of oleic acid (OA) at 150 °C under N_2_ atmosphere. After adding 15 mL of 1-octadecene (ODE), the temperature was raised to 310 °C. The composition-gradient CdZnSeS cores were synthesized by a swift injection of a Se + S stock solution (2.2 mmol of Se and S in 2.2 mL of trioctylphosphine), maintaining the reaction for 10 min. An additional 1.6 mmol of S in 2.4 mL ODE was then added for a 12 min reaction. The ZnS shell process was initiated by the rapid introduction of Zn acetate dihydrate (2.86 mmol) dissolved in a mixture of OA (1 mL) and ODE (4 mL), and the reaction was maintained at 270 °C for 10 min. To facilitate ZnS formation, 9.65 mmol of S dissolved in 5 mL was added dropwise and reacted for 20 min. The resulting green CdZnSeS/ZnS QDs were isolated and purified through successive centrifugation using a combination of solvent and nonsolvent. The resulting purified QDs were subsequently redispersed in hexane for further analysis and EML fabrication.

### 2.2. Deposition of SnO_2_ Layer by the Sputtering Method

SnO_2_ ETLs were deposited onto substrates using the radio frequency (RF) magnetron sputtering. With a base pressure maintained below 1.5 × 10^−5^ Torr, the sputtering plasma was sustained at 100 W RF power with a 10 mTorr working pressure. The layers were optimized for QLED application by adjusting the Ar/O_2_ gas flow ratio. A pre-sputtering treatment was applied for 5 min to remove potential contaminants.

### 2.3. Fabrication of the Inverted QLEDs

**Figure 1:**
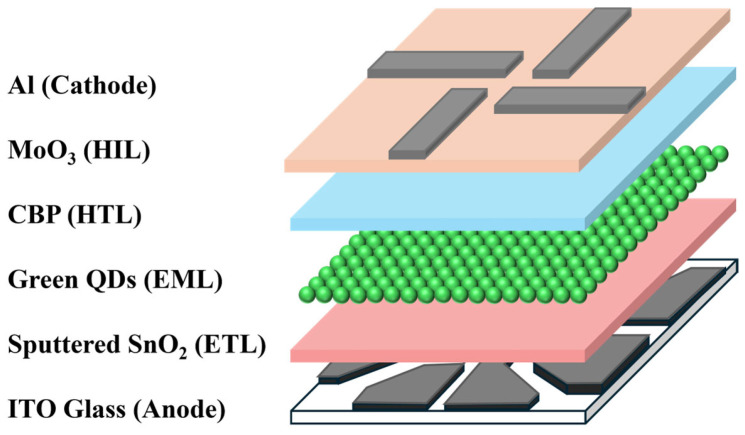
A schematic of the inverted QLEDs.

The architecture of the inverted QLEDs is schematically presented in Figure 1, with devices constructed on indium-tin-oxide (ITO) patterned glass. These substrates underwent a sequential cleaning process using isopropyl alcohol followed by deionized water. Prior to the deposition of SnO_2_ layers via RF sputtering, a 15 min ultraviolet-ozone treatment was applied to the ITO surfaces. For the EML, a QD dispersion was spin-coated onto the ITO/SnO_2_ at 5000 rpm for 20 s. To complete the device, 4,4′-bis (carbazol-9-yl) biphenyl (CBP), MoO_3_, and Al were sequentially deposited via thermal evaporation without vacuum interruption. The evaporation rates were precisely maintained at 1 Å/s for CBP, 0.5 Å/s for MoO_3_, and 3 Å/s for Al cathode.

### 2.4. Characterizations

The current density–voltage–luminance (J–V–L) parameters were evaluated using a spectroradiometer (Konica Minolta CS2000, Tokyo, Japan) coupled with a Keithley 2400 source meter (Cleveland, OH, USA) in an ambient atmosphere. These measurements allowed for a comprehensive analysis of the luminance and current efficiency profiles relative to the applied bias. By correlating the photocurrent from a silicon photodiode with the luminance measured via a spectroradiometer, we precisely determined the device’s luminance and current efficiency. The crystal phase of SnO_2_ was analyzed using an X-ray diffractometer (AERIS, PANalytical, Malvern, UK). X-ray photoelectron spectroscopy (XPS) (Thermo VG, Waltham, MA, USA) with Al Kα x-ray (E = 1486.6 eV) was used to analyze the actual chemical composition of SnO_2_. Photoluminescence (PL) characterization was performed using a PL spectrophotometer (PSI) with a fixed excitation wavelength at 365 nm. The surface morphology was measured by atomic force microscopy (AFM) (XE-100, PSIA, Suwon, Republic of Korea). The energy band structures of SnO_2_ were investigated by ultraviolet photoelectron spectroscopy (UPS) (ESCALAB 250, Thermo Scientific, Waltham, MA, USA). The operational lifetime of the QLEDs was measured using a multi-channel lifetime test system (M6000 Plus, McScience, Daejeon, Republic of Korea).

## 3. Results and Discussion

**Figure 2:**
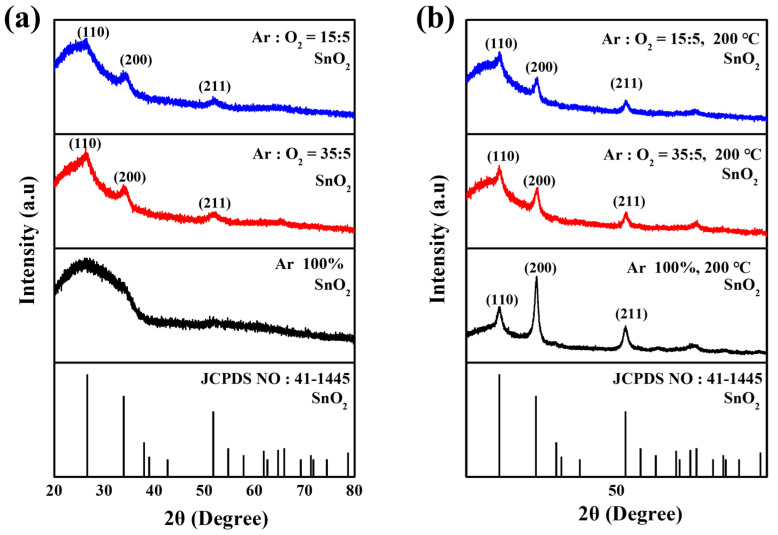
XRD patterns of sputtered SnO_2_ layer (**a**) deposited under various Ar/O_2_ ratios and (**b**) after substrate heating at 200 °C.

Figure 2a shows the X-ray diffraction patterns of the sputtered SnO_2_ layers deposited under various Ar/O_2_ ratios. All SnO_2_ films showed identical diffraction peaks at 2θ = 26.6°, 33.9°, and 51.8°, corresponding to the (110), (200) and (211) crystal planes of a rutile structure (JCPDS: 41-1445), respectively, indicating that the Ar/O_2_ ratio had a negligible effect on the fundamental crystal structure. The SnO_2_ thin films deposited in pure Ar (100%) gas exhibit an amorphous phase, which is attributed to compositional instability caused by inevitable oxygen vacancies (V_o_) during the sputtering process. However, as the O_2_ partial pressure increases during deposition, the oxygen deficiencies within the film are compensated, resulting in enhanced crystallinity. Figure 2b presents the improvement in crystallinity after the substrate heating at 200 °C. However, the temperature dependence of crystal growth diminishes with increasing oxygen partial pressure. This is attributed to the formation of compositionally stable and more ordered SnO_2_ structure at higher O_2_ partial pressures [21].

**Figure 3:**
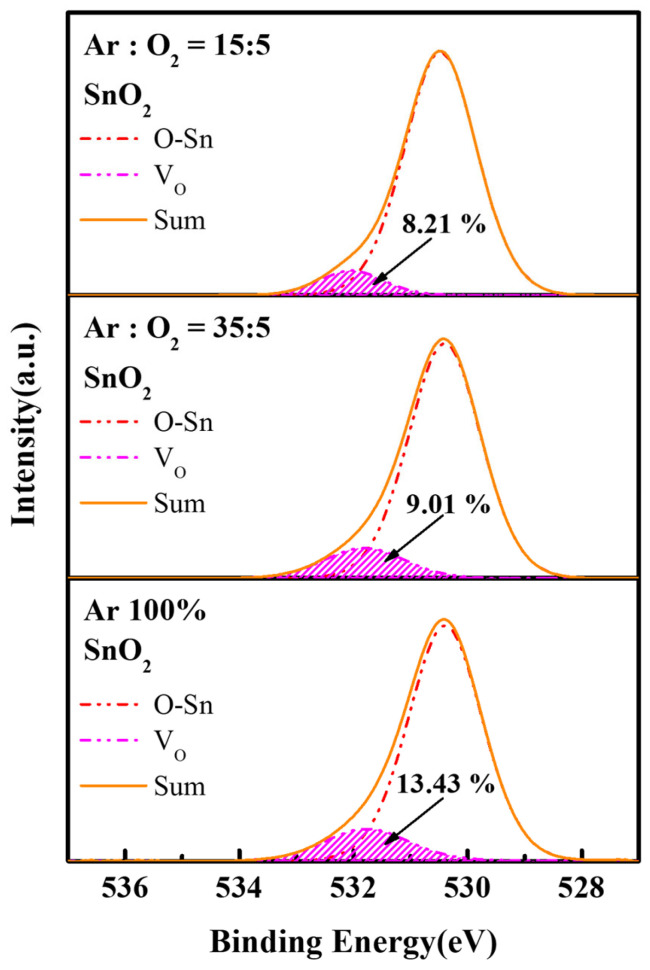
The O 1s XPS spectra of sputtered SnO_2_ films under various Ar/O_2_ ratios.

The chemical bonding states of oxygen in sputtered SnO_2_ films under different Ar/O_2_ ratios were characterized by XPS, and the resulting O 1s spectra are shown in Figure 3. The O 1s core-level spectra were resolved into two distinct components: the lower binding energy peak at 530.4 ± 0.2 eV is assigned to the Sn–O lattice bonds, while the higher peak at 531.9 ± 0.2 eV is attributed to oxygen vacancies [17]. The relative ratio of oxygen vacancies in the sputtered SnO_2_ films effectively decreased from 13.43% (Ar/O_2_ ratio = 35:5) to 8.21% (Ar/O_2_ ratio = 15:5) by increasing O_2_ partial pressure. Owing to that, oxygen vacancies serve as exciton quenching sites in QLEDs, the SnO_2_ films deposited under Ar/O_2_ ratio of 15:5, which exhibit the lowest oxygen vacancy concentration, are considered favorable as an ETL [22].

**Figure 4:**
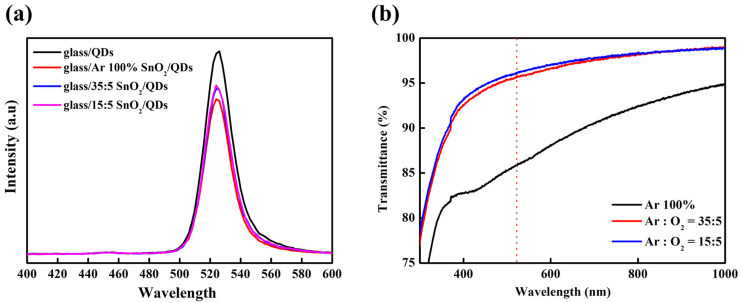
(**a**) PL spectra of QDs on various substrates: glass/QDs and glass/SnO_2_ (Ar/O_2_ ratio = Ar 100%, 35:5, 15:5)/QDs and (**b**) transmittance of sputtered SnO_2_ films under various Ar/O_2_ ratios.

Figure 4a illustrates the PL emission profiles of glass/SnO_2_/QD structures as a function of the Ar/O_2_ gas ratio used during SnO_2_ deposition. The reduced PL intensity in the glass/SnO_2_/QDs structure was attributed to exciton quenching at surface defect sites. However, PL intensities of SnO_2_ films deposited with O_2_ gas were significantly enhanced, indicating that the quenching was effectively suppressed due to reduced oxygen vacancies, as shown in Figure 3. Figure 4b presents the optical transmittance data of the SnO_2_ films under different Ar/O_2_ ratios. In a bottom-emission configuration, high optical transparency is essential because the underlying layers can unintentionally attenuate the light emitted from the QDs through absorption. The SnO_2_ films sputtered under 100% Ar gas exhibited low transmittance, whereas increasing the O_2_ partial pressure led to improved transparency due to the decreased oxygen vacancy concentration.

**Figure 5:**
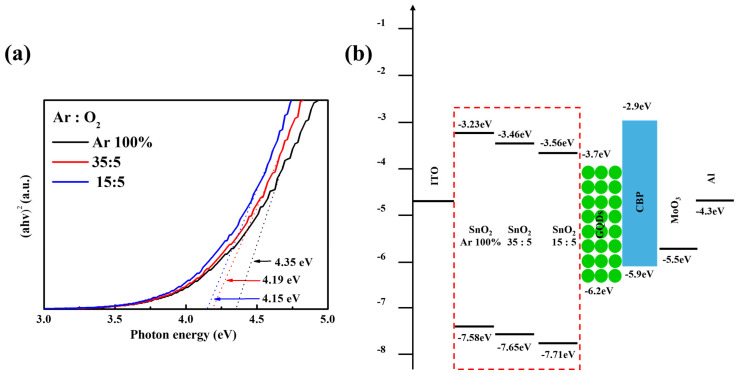
(**a**) (*αhv*)^2^ vs. *hv* plots converted from the absorption spectra of sputtered SnO_2_ films under various Ar/O_2_ ratios and (**b**) the energy band diagram of the inverted QLEDs with SnO_2_ films under various Ar/O_2_ ratios.

Figure 5a shows the absorption spectra of SnO_2_ films deposited under different Ar/O_2_ ratios, presented as the plots of (*αhν*)^2^ vs. *hν* (photon energy), where *α* is the absorbance. The bandgaps (E_g_) were estimated from the intercept of the linear portion of the absorption edge; the E_g_ values for SnO_2_ films with Ar 100%, Ar/O_2_ ratio = 35:5 and Ar/O_2_ ratio = 15:5 were 4.35, 4.19, and 4.15 eV, respectively. These data indicate bandgap narrowing upon increasing the O_2_ partial pressure, which can modulate electron injection to the EML. The energy band structures of SnO_2_ films were further investigated by UPS; the resulting secondary-electron cutoff and valence-band regions are shown in Figure S1a,b, respectively. The valence band maximum (VBM) was calculated by the incident photon energy (21.21 eV), the high-binding energy cutoff (E_cutoff_), and the onset energy in the valence-band region (E_onset_) according to the equation VBM = 21.21 − (E_cutoff_ − E_onset_) (see Table S1 for detailed values). Figure 5b presents a schematic energy band diagram of inverted QLEDs with various SnO_2_ films. Based on the calculation, the VBM positions of SnO_2_ films with Ar 100%, Ar/O_2_ ratio = 35:5 and Ar/O_2_ ratio = 15:5 were calculated to be 7.58, 7.65, and 7.71 eV below the vacuum level, respectively, exhibiting a large downshift of the VBM level with increasing O_2_ partial pressure. Based on the E_g_ and VBM values, the conduction band minimum (CBM) levels were estimated to be 3.23, 3.46, and 3.56 eV below the vacuum level for SnO_2_ films with Ar 100%, Ar/O_2_ ratio = 35:5 and Ar/O_2_ ratio = 15:5, respectively.

**Figure 6:**
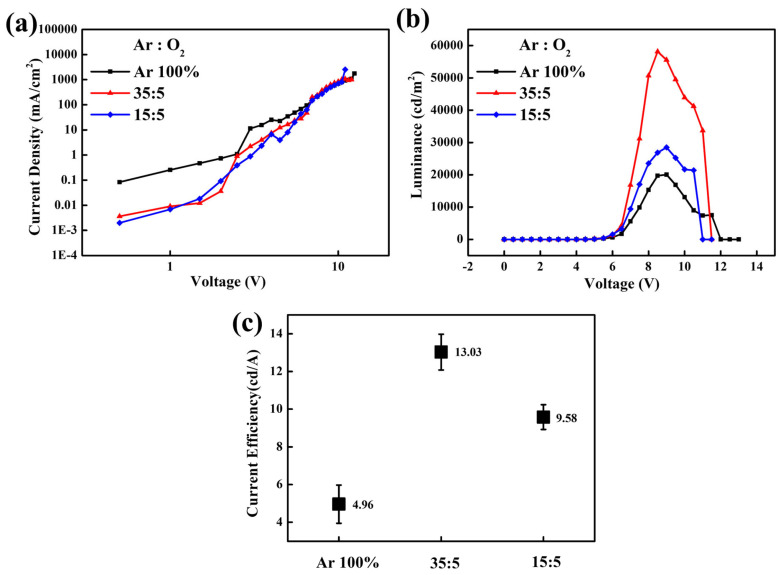
Voltage-dependent characteristics of (**a**) current density and (**b**) luminance of the inverted QLEDs with sputtered SnO_2_ films under different Ar/O_2_ ratios. Statistical data of (**c**) current efficiency for 5 devices based on various SnO_2_ films as ETL.

Figure 6 shows the voltage-dependent characteristics in luminance, current density, and current efficiency of QLEDs with various SnO_2_ films. Using the SnO_2_ films (Ar/O_2_ ratio = 35:5) as an ETL, the QLEDs exhibited a maximum luminance of 58,149 cd/m^2^ at 8.5 V and current efficiency of 13.72 cd/A at 8 V. The CBM level (−3.46 eV) of SnO_2_ films (Ar/O_2_ ratio = 35:5), which is comparable to that of green QDs, facilitates efficient electron injection and transport from the ITO into the EML, while its deep-lying VBM level (−7.65 eV) effectively blocks holes at the EML/ETL interface. We attribute enhanced performance to the downward shift of the CBM in the optimized SnO_2_ films, which facilitates electron transport by lowering the injection barrier from the electrode to the ETL, thereby improving the charge balance within the EML. The unsmooth curves in J-V graphs may originate from surface defects or oxygen vacancies of SnO_2_ thin film during the high-energy sputtering process. The sputtered thin films are denser than solution-processed ones, but these plasma-induced surface states are inevitable. Interestingly, we observed that these fluctuations were suppressed following substrate heating at 200 °C.

**Figure 7:**
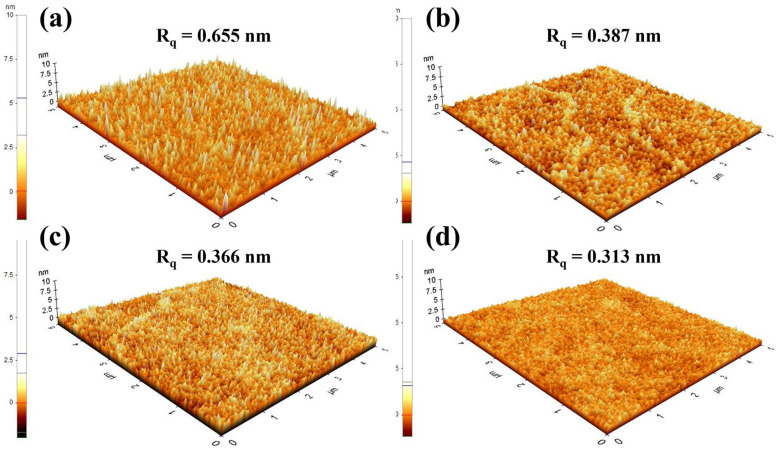
AFM images (5 μm × 5 μm) of sputtered SnO_2_ films deposited under various conditions. (**a**) Ar 100%, (**b**) Ar 100% at 200 °C, (**c**) Ar:O_2_ = 35:5, and (**d**) Ar:O_2_ = 35:5 at 200 °C.

**Figure 8:**
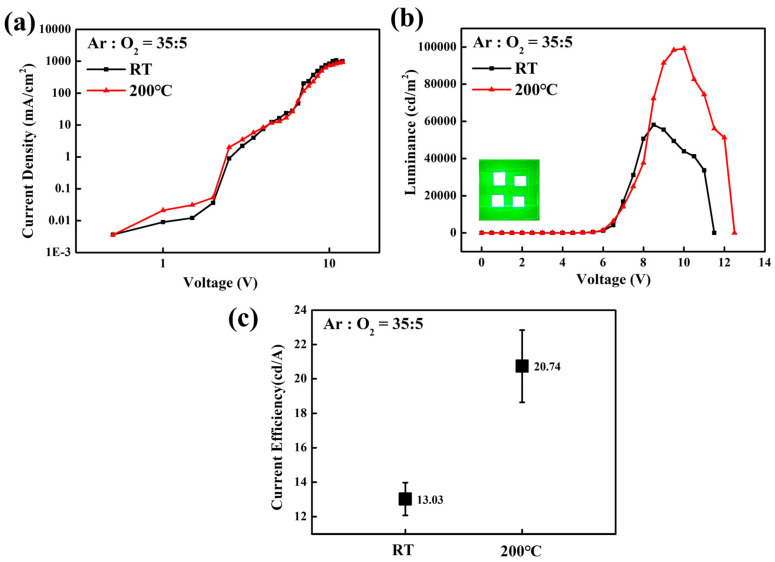
Voltage-dependent characteristics of (**a**) current density and (**b**) luminance of the inverted QLEDs with sputtered SnO_2_ films after substrate heating at 200 °C. The inset shows the green emission from 2.5 mm × 2.5 mm pixels. Statistical data of (**c**) current efficiency for 5 devices based on substrate heating at 200 °C.

While the optimization of the Ar/O_2_ ratio during the sputtering process successfully controlled the stoichiometry and oxygen vacancy of the SnO_2_ films, the surface morphology is another critical factor determining the performance of QLEDs [23,24]. Sputtered films deposited at room temperature often exhibit rough surfaces, which can lead to poor interfacial contact with the QD EML. The surface profiles of various SnO_2_ films were measured using AFM to observe morphological changes induced by different Ar/O_2_ ratios and substrate heating at 200 °C. As shown in Figure 7, the surface roughness decreased with increasing oxygen partial pressure and substrate heating, which is mainly attributed to the formation of a dense and uniform surface facilitated by improved adatom mobility [25]. Consequently, the QLEDs with SnO_2_ films (Ar/O_2_ ratio = 35:5) with substrate heating at 200 °C exhibited improved device performance, as shown in Figure 8, achieving a peak luminance of 99,212 cd/m^2^ at 10 V and current efficiency of 21.17 cd/A at 8.5 V.

**Figure 9:**
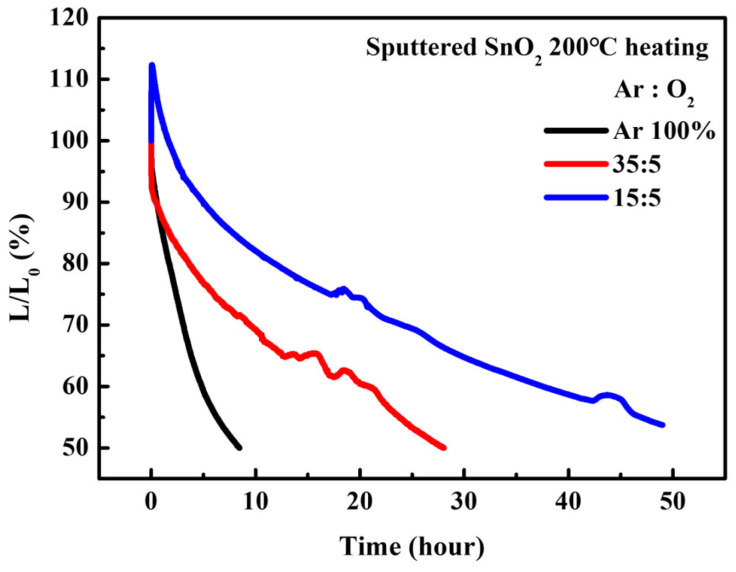
Operational lifetime characteristics of QLEDs with various sputtered SnO_2_ ETLs.

Interestingly, although the maximum luminance and current efficiency of the QLEDs with SnO_2_ films (Ar/O_2_ ratio = 15:5) were lower than those of the QLEDs with SnO_2_ films (Ar/O_2_ ratio = 35:5), they exhibited excellent operational stability. As shown in Figure 9, the predicted T_50_ at 100 cd/m^2^ was 4480 h, based on the T_50_ at 1000 cd/m^2^ with an acceleration factor of 1.9. Excitons are easily quenched at defect sites via charge transport at the SnO_2_/QD interface; this quenching process is effectively suppressed by the reduction of oxygen vacancies, as confirmed in Figure 3.

Although spin-coating is commonly employed for inorganic ETL fabrication, RF sputtering facilitates superior control over physical and chemical properties of deposited films. Our study reveals that the device efficiency is highly sensitive to the CBM position, which dictates the charge injection balance. Additionally, the reduction of oxygen vacancies was identified as a fundamental mechanism for enhancing long-term stability through the prevention of exciton quenching. Although substrate heating suppressed the J–V fluctuations, the luminance drop at high current densities persisted. This suggests that while structural defects were partially removed, the device roll-off phenomenon is likely governed by charge imbalance. Future works involving interfacial modification or high-mobility hole transport materials could further improve the charge balance and minimize exciton quenching, leading to more stable luminance under high-current operation.

## 4. Conclusions

This study demonstrates the successful application of SnO_2_ thin films as ETLs in QLEDs, with properties tailored by Ar/O_2_ ratios. The analysis confirmed that increasing O_2_ content optimizes the CBM position, thereby enhancing electron transport from the cathode. QLEDs with SnO_2_ films (Ar/O_2_ ratio = 35:5) afforded a maximum luminance of 99,212 cd/m^2^ and current efficiency of 21.17 cd/A after heating at 200 °C. Additionally, the stability of devices was improved by reducing the oxygen vacancies of SnO_2_. Based on the improved device characteristics, the optimized SnO_2_ films are highly effective inorganic ETL materials, offering significant advantages for stable and efficient QLEDs.

## Data Availability

The data presented in this study is available on request from the corresponding author.

## Supplementary Materials

The following supporting information can be downloaded at https://www.mdpi.com/article/10.3390/nano16010031/s1, Table S1: Energy levels of sputtered SnO_2_ films under various Ar/O_2_ ratios; Figure S1: UPS spectra of (a) the secondary-electron cutoff and (b) valence-band edge regions of sputtered SnO_2_ films under various Ar/O_2_ ratios.
