# Supplementary material for: Highly Efficient and Stable Quantum Dot Light-Emitting Diodes Employing Sputtered SnO2 Layer as Electron Transport Layers
Source: Nanomaterials (Basel). 2025 Dec 25;16(1):31. doi: 10.3390/nano16010031 (PMC12788106; doi:10.3390/nano16010031)
Supplement: Supplementary file 1 [file nanomaterials-16-00031-s001.zip › nanomaterials-4049405-supplementary.pdf]

Supplementary materials

Highly Efficient and Stable Quantum Dot Light-Emitting Diodes by Employing Sputtered SnO_2 Layer as Electron Transport Layers

Jaehwi Choi and Jiwan Kim *

Department of Advanced Materials Engineering, Kyonggi University, 16227, Republic of Korea

Table S1. Energy levels of sputtered SnO_2 films under various Ar/O_2 ratios.

Sputtered SnO_2	SECO (eV)	Val. edge (eV)	Bandgap (eV)	VBM (eV)	CBM (eV)
Ar 100%	16.83	3.2	4.35	7.58	3.23
Ar : O_2 = 35:5	16.81	3.25	4.19	7.65	3.46
Ar : O_2 = 15:5	16.79	3.29	4.15	7.71	3.56

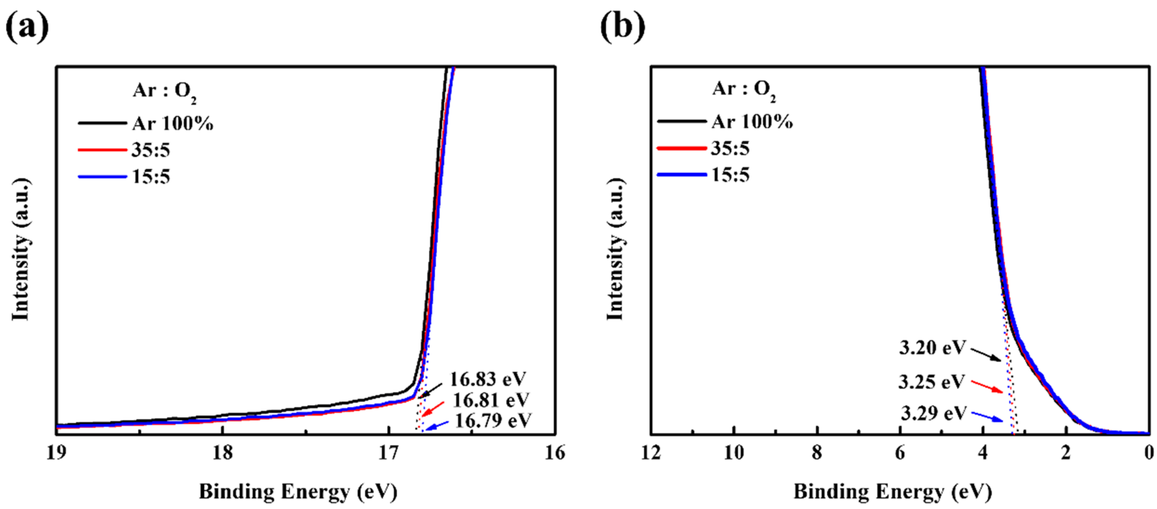

Figure S1. UPS spectra of (a) the secondary-electron cutoff and (b) valence-band edge regions of sputtered SnO_2 films under various Ar/O_2 ratios.
